# Evaluation of massive education in prison health: a perspective of health care for the person deprived of freedom in Brazil

**DOI:** 10.3389/fpubh.2023.1239769

**Published:** 2023-08-23

**Authors:** Janaína L. R. S. Valentim, Sara Dias-Trindade, Eloiza S. G. Oliveira, Manoel H. Romão, Felipe Fernandes, Alexandre R. Caitano, Marilyn A. A. Bonfim, Aline P. Dias, Cristine M. G. Gusmão, Philippi S. G. Morais, Ronaldo S. Melo, Gustavo Fontoura de Souza, Kelson C. Medeiros, Maria C. F. D. Rêgo, Ricardo B. Ceccim, Ricardo A. M. Valentim

**Affiliations:** ^1^Laboratory of Technological Innovation in Health (LAIS), Federal University of Rio Grande do Norte (UFRN), Natal, State of Rio Grande do Norte, Brazil; ^2^Centre for Interdisciplinary Studies, University of Coimbra, Coimbra, Portugal; ^3^Department of History, Political and International Studies (DHEPI), Faculty of Arts, University of Porto, Porto, Portugal; ^4^Institute of Human Formation With Technologies, State University of Rio de Janeiro (UERJ), Rio de Janeiro, Brazil; ^5^Oswaldo Cruz Foundation (FIOCRUZ), Rio de Janeiro, Brazil; ^6^Department of Biomedical Engineering, Federal University of Pernambuco, Recife, Brazil; ^7^International Council for Open and Distance Education, Oslo, Norway; ^8^Postgraduate Program in Education, Federal University of Rio Grande do Norte (UFRN), Natal, State of Rio Grande do Norte, Brazil; ^9^Postgraduate Program in Education, Federal University of Rio Grande do Sul (UFRGS), Porto Alegre, State of Rio Grande do Sul, Brazil

**Keywords:** prison health, prison system, health education, health education in prison system, health massive education, evaluation of health massive education, public health

## Abstract

Education, with an emphasis on prison health, has acted as a policy inducing changes in work processes, which the Brazilian National Health System (SUS) has used, and which is present in permanent health education, which promotes health care for people deprived of liberty. This article aims to present an analysis of the impacts of the strategy of massive education on prison health in Brazil from the perspective of health professionals and other actors operating in the Brazilian prison system. The data used in the study come from a questionnaire consisting of 37 questions applied nationwide between March and June 2022. Responses were collected from students who completed the course “Health Care for People Deprived of Freedom” of the learning pathway “Prison System”, available in the Virtual Learning Environment of the Brazilian Health System (AVASUS). This course was offered nationally, whose adhesion (enrollment) occurred spontaneously, i.e., the course was not a mandatory. The data collected allowed us to analyze the impacts of massive education on prison health. The study also shows that the search for the course is made by several areas of knowledge, with a higher incidence in the health area, but also in other areas, such as humanities, which also work directly with the guarantee of the rights of people deprived of liberty, which are professionals in the areas of social work, psychology, and education. The analysis based on the data suggests that the massive education mediated by technology through the courses of the learning pathway, besides disseminating knowledge–following the action plan of the 2030 Agenda of the United Nations Educational, Scientific and Cultural Organization (UNESCO)–, are an effective tool to promote resilience in response to prison health and care demands of people deprived of liberty.

## 1. Introduction

Prison health is an emerging agenda in Global Health, which has been strengthened by the World Health Organization (WHO) and the Pan American Health Organization (PAHO) (1–5). In Brazil, prison health efforts are coordinated by Brazil's Ministry of Health (MoH) and the National Council of Justice (CNJ) in order to implement the National Comprehensive Health Care Policy for People Deprived of Liberty (PNAISP) and the Program of Intersectoral Actions of Health Care and Social Assistance for the Prison System (PAISA) (1, 6, 7).

Worldwide, the population deprived of liberty exceeds 10 million people, with an average growth rate of 20% per year, i.e., higher than the estimated percentage increase in the general population (18%), data observed since 2000. For every 100,000 population, there are 144 people in prison, according to United Nations (UN) data (8, 9). The growth or decline in the prison population varies by country or region. In Oceania, for instance, there was a 60% increase, while in the Americas it was 40% in contrast to Europe, which showed a 21% drop in the prison population (8).

In Brazil, the prison population exceeds 800,000, with 392 incarcerated people per 100,000 population (10, 11). With the prevalence of diseases, mainly sexually transmitted infections (STIs) and tuberculosis, health in prison settings has become a public health emergency (12–15). Therefore, several strategies and public policies directed at the prison system were adopted in order to tackle this public health crisis in the country. The Penal Execution Law (LEP) No. 7,210 of July 11, 1984, is a milestone (11). This law addresses the rights of people deprived of freedom in the Brazilian prison system, being essential for their social reintegration. However, there is still much work to be done to effectively guarantee the rights of this population, which is considered vulnerable, especially regarding health services (1, 16).

Prison health, situated as a human right, in the sense of the universal right to health, cannot be overlooked because its effects, in addition to affecting this specific population, influence the health of the community, and consequently, public health (1, 4, 11, 17). Therefore, it is essential to strengthen comprehensive and universal health, which can be developed through effective public policy interventions aimed at reducing social inequalities and promoting equity, in line with the goals of the UN's 2030 Agenda for Sustainable Development (18–22). This agenda sets forth 17 Sustainable Development Goals (SDGs) for the planet and society (1, 23, 24).

As the prison population increased, prison health became a commitment of the United Nations Educational, Scientific and Cultural Organization (UNESCO), therefore under the action plan of the UN's 2030 Agenda. Also committed to this plan are the WHO, PAHO, and Brazil's MoH, which highlight the importance of improving the prison system and health practices to fully reach incarcerated people, prison officers, and the surrounding community (1, 25–28).

Intending to face the public health crisis in the prison system, and based on legal principles, the Brazilian MoH has tailored public policies for the Brazilian Prison System. Therefore, it is always acting to insert the theme in the national health agenda.

Education, with emphasis on prison health, acts as a policy inducing changes in work processes, which SUS has used, and which is present in the permanent education of health professionals (29–31). However, in Brazil, a country with continental dimensions and great social, cultural, and regional diversity, permanent health education poses a great challenge. In this vein, the country requires health education strategies that can effectively respond to health services demands and public health crises (1, 32–35).

Brazil has 26 federative units (FUs) and one Federal District, 5,700 municipalities, 200,000 health facilities distributed throughout the country, and more than 3.5 million health professionals, who work in the most diverse areas and levels of health care–primary care, secondary/specialty care, and tertiary/hospital care (36–40). These factors are determining factors for the organization and structuring of continuing education for workers in the SUS. Especially, in scenarios of public health crisis, such as the Brazilian syphilis epidemic in 2016 and the COVID-19 pandemic in 2020 (1, 32, 41). Situations and context that require the MoH to implement strategies based on massive health education through technological mediation, which also includes the provision of courses developed from the perspective of self-learning (35, 42–46).

In the context of massive health education, one of the strategies adopted by the MoH is the Virtual Learning Environment of the Brazilian Health System (AVASUS). AVASUS is an online, free, and open knowledge educational platform in health (42, 47–50). In 2023, AVASUS recorded more than 2.6 million enrollments in more than 400 courses, which accounts for nearly 10,000 hours of online educational offerings. Recent studies show the relevance of this platform as a tool to induce public health policies. It has been an important instrument in the process of education and learning of health workers in Brazil because it has directly contributed, and in scale, to the improvement and supply of health services in the country (41, 51, 52). This massive education process, through AVASUS, has resulted in changes in work processes at various levels of health care, which has promoted resilience in the system and timely response to health problems (32, 41, 47, 51, 53).

In 2016, the MoH declared the epidemic of syphilis due to the significant increase in the number of cases nationwide (54–56). In line with previous studies and based on the recommendations from the Brazilian Federal Court of Accounts (TCU), the MoH and PAHO have recognized that populations considered as vulnerable or key have high prevalence and incidence of STIs, in addition to other diseases, e.g., tuberculosis. In Brazil, the prison population is considered a vulnerable population, therefore, with the support of the “Syphilis No!” Project (SNP), the learning pathway “Prison System” was elaborated and made available on AVASUS, thus addresing topics related to prison health (15, 21, 57–60). Composed of four educational modules, this learning pathway has surpassed 30,000 students enrolled from the five Brazilian regions (61).

A study of an educational module of this pathway, the course “Health Care for People Deprived of Freedom”, related massive education with technological mediation to the increase in syphilis testing and diagnosis in the Brazilian prison system. The results highlighted the relationship between continuing health education and work process changes (1). A limitation of the study was the use of only secondary data, lacking in this study, an analysis based on data that could observe the perception of health professionals and the relationship to the impacts of this training process on prison health.

Considering this gap, this study analyzes the impacts of the strategy of massive health education on prison health in Brazil, from the perspective of health professionals and other workers operating in the Brazilian prison system.

## 2. Materials and methods

Our study started with the following research question Q1: “How has massive education, mediated by technology, contributed to Brazilian prison health?” Therefore, the materials used and the methodology applied for the analysis of the impacts of massive education on prison health from the perspective of health professionals were structured and organized to subsidize the answer to this research question.

The data collected for the studies come from a questionnaire that was applied nationally, during the period from 03/23/2022 to 06/30/2022 to students who completed the course “Health Care for People Deprived of Freedom”. This course was chosen as the target, for being the first to be offered in the learning pathway, and also because, at the time the questionnaire was applied, it was the only course with a significant number of students in all regions of the country (>1,000 students in all five regions), that is, able to answer the questionnaire. As the course “Health Care for People Deprived of Freedom”, also, was part of the Postgraduate Program in Family Health Strategy (PEPSUS), it had already been offered since 2018 in AVASUS and had health professionals as its target audience, which was the main focus of our questionnaire. As the offer period was longer, from 06/07/2018 to 03/23/2022, this course had already reached an expressive number of enrolled students and completers, unlike the other three courses of the learning pathway, which had been launched in December 2021, i.e., a few months before the application of the questionnaire.

The AVASUS technical-administrative team applied the questionnaire to all students completing the course. Therefore, during the questionnaire application period, 6,345 students who had completed the course were eligible to answer it. Of these 6,345 students, 270 answered the questionnaire, even though it was not mandatory to do so. The expected sample size would be 184 responding graduate students (participants) so that the degree of confidence in this research would be 90% with a margin of error of 6%. In this case, the questionnaire was answered by 86 more graduating students than expected, which is 46.73% more. The sample size was determined by the model described in Equation 1.


(1) $$\begin{array}{l} n=\frac{NZ^{2}\;p\left(1-p\right)}{\left(N-1\right)e^{2}+Z^{2}\;p\left(1-p\right)} \end{array}$$

where,

*n*: Size of the sample to be calculated;
*N*: Universe size (e.g. 6,345 final-year students);
*Z*: Deviation from the mean value that is accepted to reach the desired confidence level. Depending on the confidence level that is sought, a certain value should be used, which is given by the shape of the Gauss distribution. The most frequent values are (in bold the value used to determine the confidence level for this research):

- 90% confidence level, *Z* = 1.645;
- 95% confidence level, *Z* = 1.96;
- 99% confidence level, *Z* = 2.575.

*e*: Is the maximum margin of error that you want to admit (e.g. 6%); and
*p*: Is the confidence proportion that is expected to be found.

The questionnaire was composed of 37 questions, in the following formats: (1) multiple choice (more than one possible answer), (2) objective (only one possible answer), and (3) open (where the student participant could answer in free text). The questionnaire can be found in the Zenodo (62) or AVASUS (63) repository. The questions were divided into six dimensions:

1. Student Profile: six questions;
2. Knowledge Sharing: eight questions;
3. Content: eleven questions;
4. Right to health of persons deprived of liberty: six issues;
5. Professional practice: two questions;
6. Workplace: four questions.

The questionnaire was composed of questions with the following characteristics: nominal categorical, ordinal categorical, Likert (64), and open (free text) questions, as described below:

I. Student profile: nominal categorical questions;
II. Knowledge sharing: nominal categorical questions;
III. Content: Ordinary questions with five items;
IV. Right to the health of persons deprived of liberty: common questions;
V. Professional practice: two Likert questions and four open questions;
VI. Workplace: four Likert questions and two open questions.

After development, the questionnaire was reviewed and improved by a team of experts, with more than ten years of experience, as described in Table 1, in education, health education, prison health, and the prison system.

**Table 1:** Profile of the experts who reviewed the questionnaire.

**Specialist**	**Expert profile description**
01	University professor, sanitarian, master in education, PhD in Clinical Psychology, and post-doctorate in Medical Anthropology. Specialist for over 30 years in Health Education.
02	University professor, nurse, master in Family Health, and doctor in Collective Health. Specialist in Public Health for over 11 years.
03	University professor, nurse, specialist in family health, Master in Public Health, and PhD in Collective Health. Develops research in the area of Prison Health for over 12 years.
04	University professor, master in Social Psychology, and doctor in Public Policies and Human Formation. Specialist in Prison System for over 18 years.

All data from the questionnaire, before being used in this research, were anonymized by a technical team responsible for AVASUS administration. Since it is a questionnaire of interest to AVASUS management, the data from its application were of public and administrative interest. The purpose of the questionnaire was to evaluate and improve the quality of the educational modules offered on AVASUS. Since it is public domain information, which can also be used for various purposes, the anonymized data were published on AVASUS and can be accessed in a free and open-access repository: In addition, a data dictionary (meta-data) and the developed questionnaire were included in this repository as a way to contribute to other analyses and studies (62). Therefore, this research used a free, open, and public domain database, whose information was aggregated without the possibility of individual identification. According to these characteristics and based on Resolution 674/2022 of the National Health Council (CNS, acronym in Portuguese) of the MoH, this research is exempt from registration with the Committee for Ethics in Research (CEP)/Brazil.

## 3. Results

In order to evaluate the effectiveness of health education, it should be recognized that the education-health dyad constitutes an epistemic field of significant relevance for public health policy formulation (29, 65, 66). Hence, examining and analyzing such dimensions in the context of the SUS workforce and their effects on health services and public health are indispensable steps to assess the resilience of work processes–i.e., of professional practice–and, therefore, health system resilience (1, 32, 37, 41, 67, 68). In addition to purely examining the field of health education, which considers quality aspects of training, content, course, and educational model, it is necessary to take into account its context, i.e., the impacts of this education on health services (69, 70).

In this vein, with the intention of evaluating the impacts of health education that has been massively implemented in prison health in Brazil, this section presents the data obtained from the processing and analysis of the questionnaire responses. To improve understanding and facilitate reading, the information was grouped under the questions applied. Consequently, a description was not made for each question on the questionnaire - the findings, for the most part, were described in grouped form in graphs and or tables.

**Figure 1:**
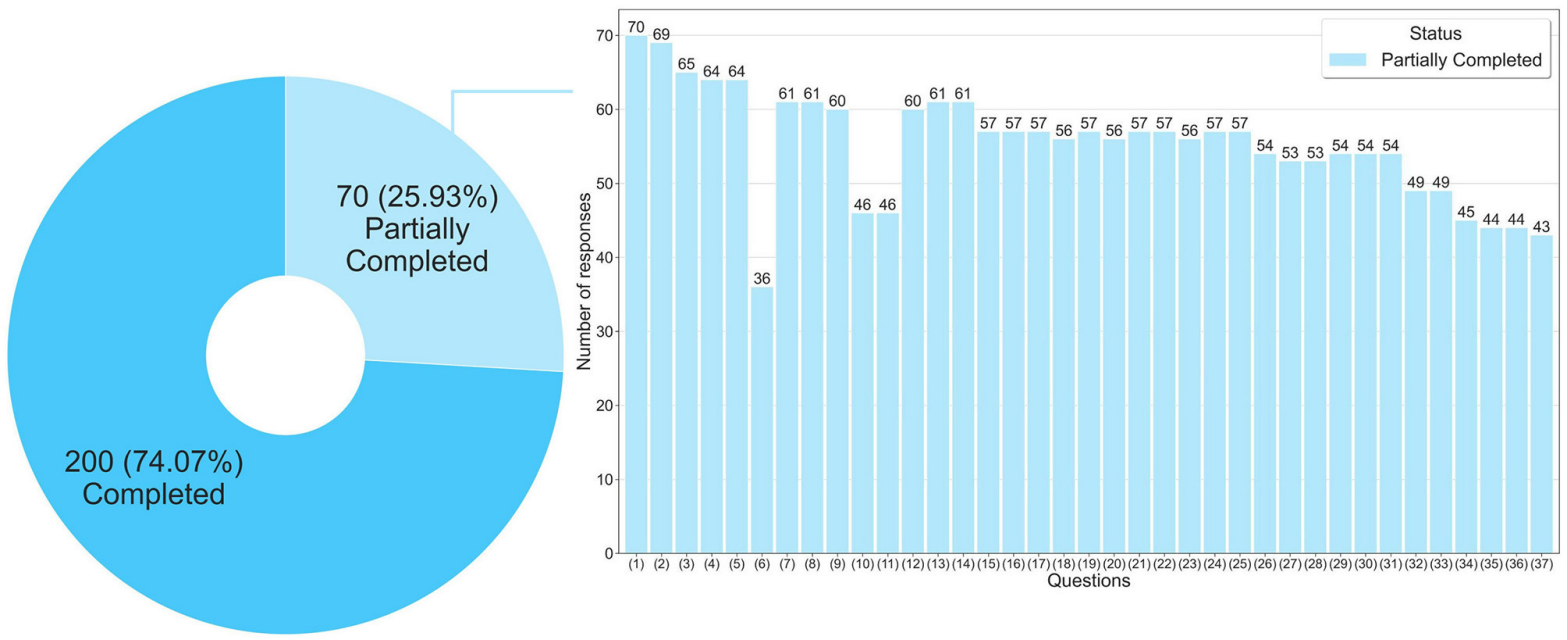
Frequency of responses per question in the questionnaire.

The first data, presented in Figure 1, shows that 200 final-year students answered all the questions in the questionnaire and 70 answered partially. In Figure 1 it is also possible to verify the frequency of answers per question, for example, questions 1 and 6 had the highest and lowest number of answers, respectively. It is important to note that no question was answered by all students, and also no question was left unanswered, which means that there was no bias in the responses obtained, for example, students did not all mark the same question or all failed to mark a single question.

Regarding the question “When you took the course, were you a…?”, which tries to find out what the professional performance of the respondent was when he/she took the course “Health Care for People Deprived of Freedom”. The answers given to this question allowed us to verify that 143 respondents were already health professionals when they took the course, i.e., ~48%. It is noteworthy in the response to this same question, that 137 (respondents) were health students when they took the course, ~46% of all respondents. Health workers and students interested in the course topic accounted for nearly 94% of respondents. This is a significant result, as it allows us to visualize the course's reach and the massive education model adopted accordingly to the target audience. It is important to emphasize that this course is not a mandatory recommendation, so this reach occurred spontaneously and naturally.

**Figure 2:**
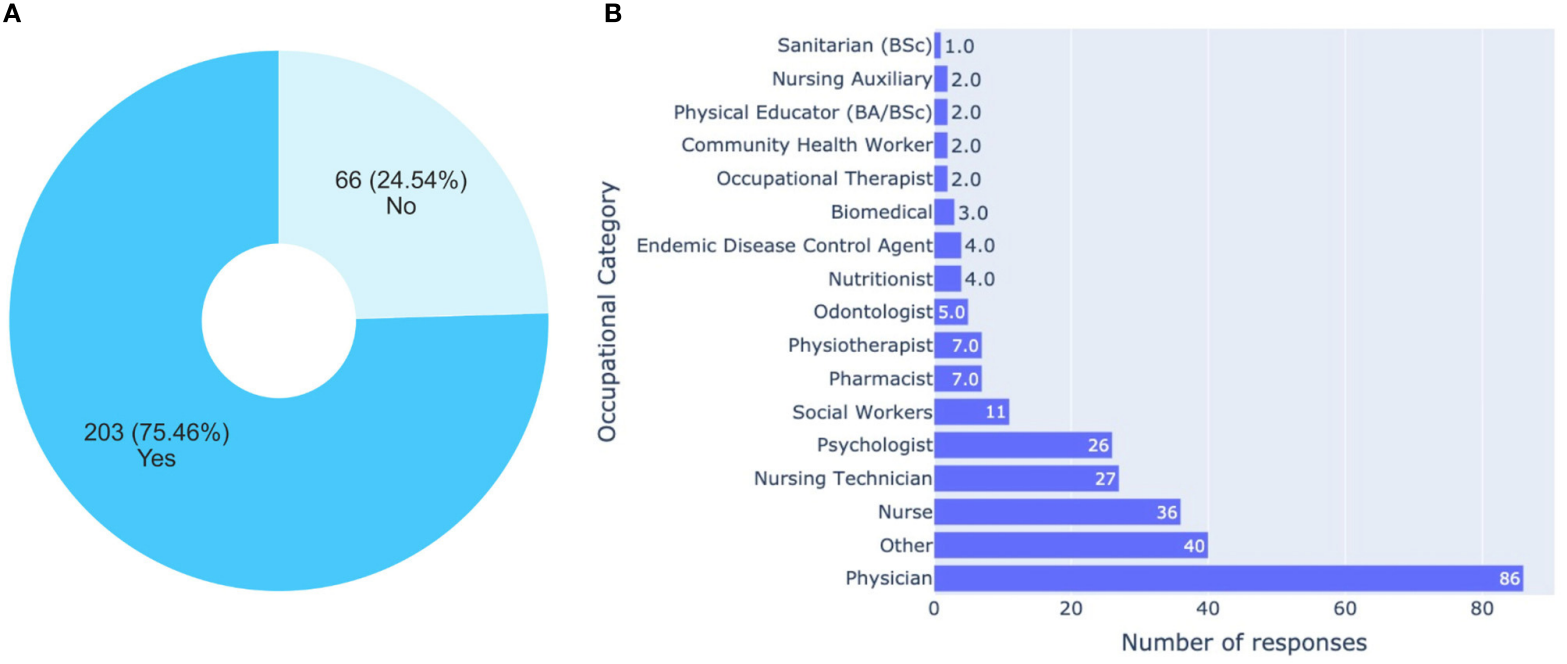
Analysis related to the occupation. **(A)** Are you currently working as healthcare professional? **(B)** If you work as a health professional, select the alternative referring to your occupational category.

Figure 2 displays a chart referring to the professions of the respondents. It mainly focuses on the health professions, which constitute the majority of the course's audience. It shows a predominance of physicians and nurses, with 86 and 36, respectively. These two professions added together, represent 60.09% of the professions of those who answered the questionnaire. This data draws attention because in Brazil it is common that the most significant spontaneous adhesion to open courses is usually by nurses, as they are the largest workforce in the health area (2,822,661 nurses in all levels, e.g., graduates, technicians, and aides), especially when compared to doctors (564,385 active doctors) (71, 72).

When asked “In which area of the health sector are you currently working?”, 132 (42.31%) responded that they worked in primary health care. Also related to this question, more than 21% of the respondents reported that they worked in the area of training or education. One explanation for this is that in Brazil there is an incentive for educators to work in the prison system because incarcerated people can have their sentences reduced by attending classes. However, further research would be needed to better understand this phenomenon (73).

As for the respondents' performance in prison health, the data from the question “Do you work or have you worked in the prison system?” showed links to all types of Prison Primary Care Teams (eAPP), teams established in Brazilian legislation (74). However, the highest incidence is for the eAPP-I type. This means that almost 80% of the respondents informed that they work or have worked in prison units that contain up to 300 (three hundred) detainees, with a health service that works six hours a week. When added to the respondents who worked or work in an eAPP-I with Mental Health (Complementary Psychosocial Team of Prison Primary Care), this percentage increases to ~90%. Regarding the eAPP-II, 2.54% of the students answered that they had worked or work in prison units that contain from 101 (one hundred and one) to 1,700 (one thousand and seven hundred) deprived of freedom, with a health service that works 20 (twenty) hours a week. When Mental Health was included in this type of prison primary care team, this percentage increases to 3.39%. Following the eAPP-III, 5.93% of the respondents informed that they worked or work in prison units with health services in a 30 (thirty) hours a week routine, that contains from 1,201 to 2,700 people deprived of freedom. This finding enhances our perception as it shows that the course reached not only health professionals but most health professionals who work or have worked in the prison system. Regarding the prison system, a notable aspect is when we have the intention to improve and enhance health services in the prison system.

According to the answers to the question “Why did you choose the course?”, 73.05% answered for the content, 20.78% for the educational model (self-learning), and 6.17% for other issues. When most of the respondents (225) stated that they chose the course for the content, they evidenced that there is a need for training in this area and that the content was adequate to the demands of prison health. It is important to highlight that there is a shortage of qualified material for prison health training. Regarding the 64 respondents who said they chose the course because of the educational model, self-learning, this is justified by the high workload to which health professionals are submitted, therefore, many of these professionals do not have time to attend classes in the face-to-face model. Therefore, health professionals need more flexible models of permanent and continuing education. In this case, this was possible through a self-instructional course with technological mediation.

When the respondents were asked about the main reason for taking this course, 74.41% (highlighted in bold) said they did so because of the direct or indirect relationship it establishes with their work activities. This percentage reinforces the scope of the massive education among the target public of interest in the course. Among the answers, the students listed the following motivations:

1. The need to further my education (25.24%);
2. The relationship it establishes with my professional activity (19.59%);
3. For the opportunity to access unknown content (15.44%);
4. For certification (12.24%);
5. Meet the demand of the health facility where I work (9.79%);
6. For functional progression (7.91%);
7. For the duration (workload) (3.58%);
8. The referral of co-workers (2.64%);
9. Other reason (1.88%);
10. Due to publicity about the course I saw (1.69%).

**Figure 3:**
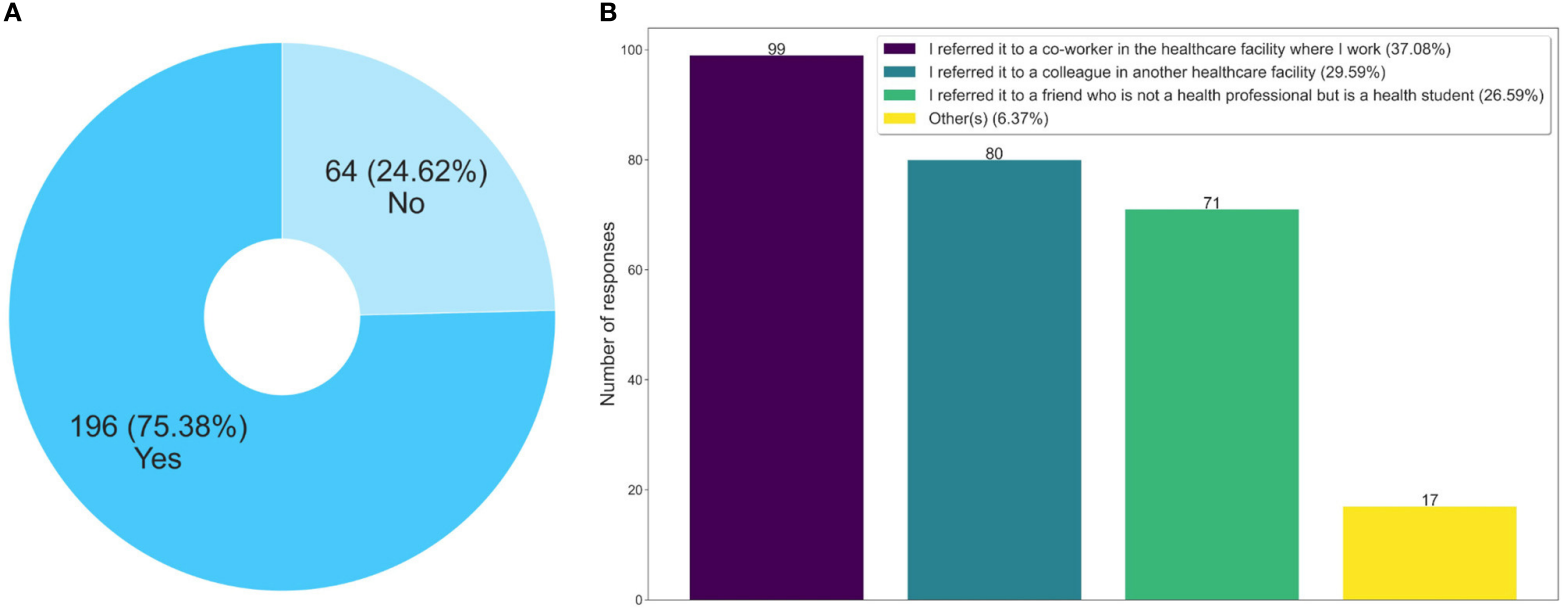
Analysis related to recommend the course to other people. **(A)** Did you recommend this course? **(B)** To whom did you recommend this course?

Regarding referring the course to others, two questions were asked; the first is whether the respondent referred the course and the second is to whom they referred the course, represented in Figures 3A, B. Note that another 75% of the respondents stated that they referred this course to other people. Of these, 66.67% referred the course to other health professionals in the facility where they work, or in another facility. The referral of the course to co-workers helps to explain the level of capillarity and the speed of the reach of the massive open training, through technological mediation in a continental territory such as Brazil in a thorny and still little-known theme in the world that is prison health.

Besides these questions, an interesting finding was that 26.59% of the respondents indicated this course not for a health professional, but for a student. This data is relevant, as it shows that a process of improvement of future health professionals, who may work in prison health, is also taking place, something not yet observed in the traditional disciplines of health courses.

For 82% of the respondents, the main reasons they indicated the course to other people were the content (44.59%), the methodology (20.10%), and the self-instructional model (17.53%), according to the data from the answers to the question “Why did you recommend this course?” It is well known that health professionals are in great demand for the services and care of patients. Generally, these professionals have more than one employment relationship, therefore, their workload is quite compromised (75). This labor characteristic, related to health professionals, can make it unfeasible to develop their educational activities in the face-to-face format, which helps to explain these cited percentages.

**Figure 4:**
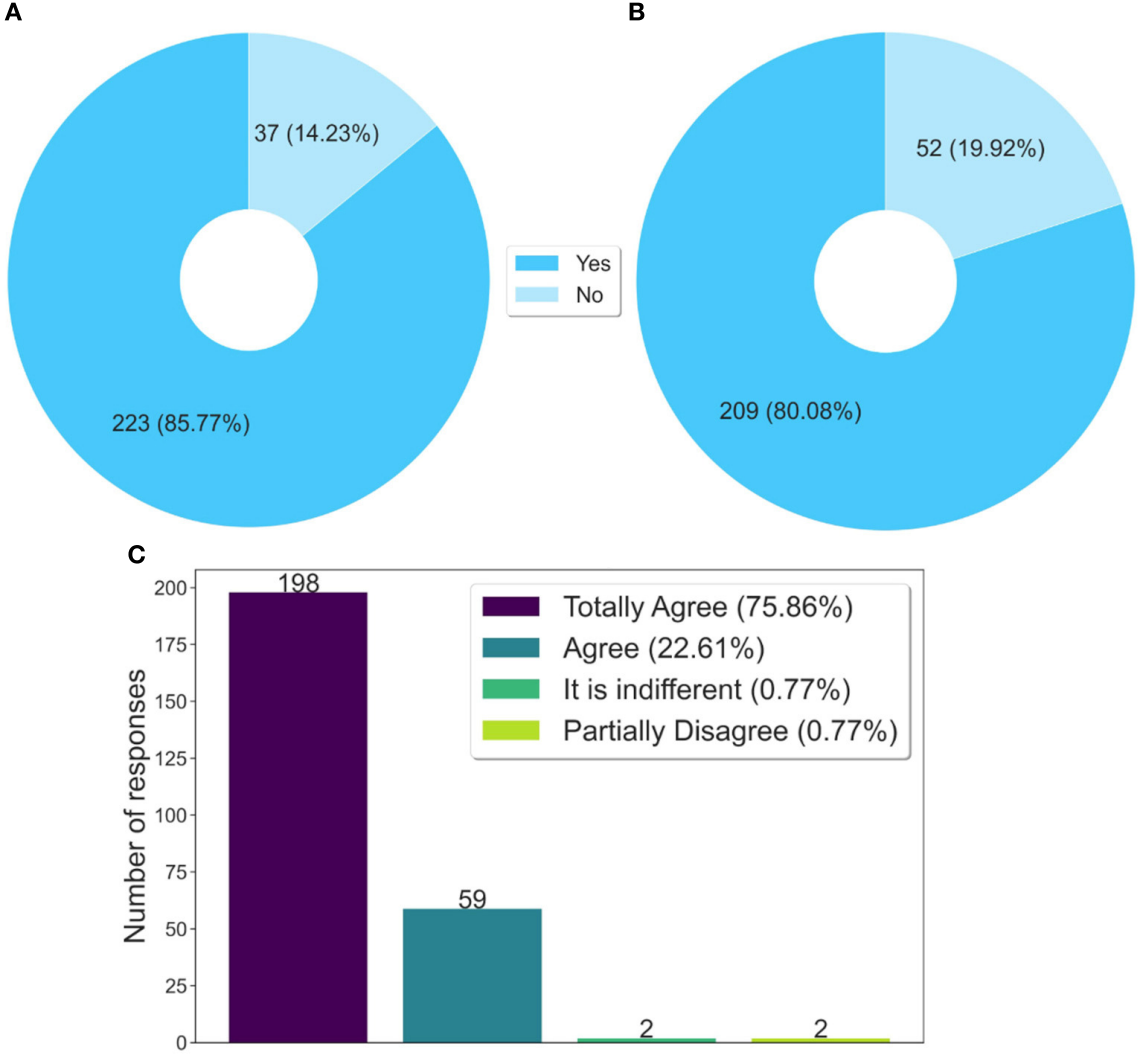
Analysis of knowledge sharing. **(A)** Have you shared what you learned in the course with your co-workers? **(B)** Did you share what you learned in the course with your family and community? **(C)** Is the course content important to be shared?

According to the data presented in Figure 4, the students who answered the questionnaire said they shared what they learned with their work colleagues and also with the family and the community, respectively 85.77% in Figure 4A and 80.08% in Figure 4B. The sharing of learning with co-workers is an important finding, as it shows that the training given is reaching other health professionals who did not take the course. Therefore, it is a transversal form, which occurs through the dissemination of knowledge from the interactions at work, i.e., in the health service, an aspect that contributes to indirectly expanding the training spectrum.

Another fact that reinforces the above, still in the same Figure 4C, is that 98.47% (almost 100%) of the respondents agreed that the course content is important, and should be shared. This data shows that the contents of this course have meaning, that is, they make sense to the students, particularly to those who work in prison health. Another aspect is that natural or spontaneous sharing is also an indicator of quality.

**Figure 5:**
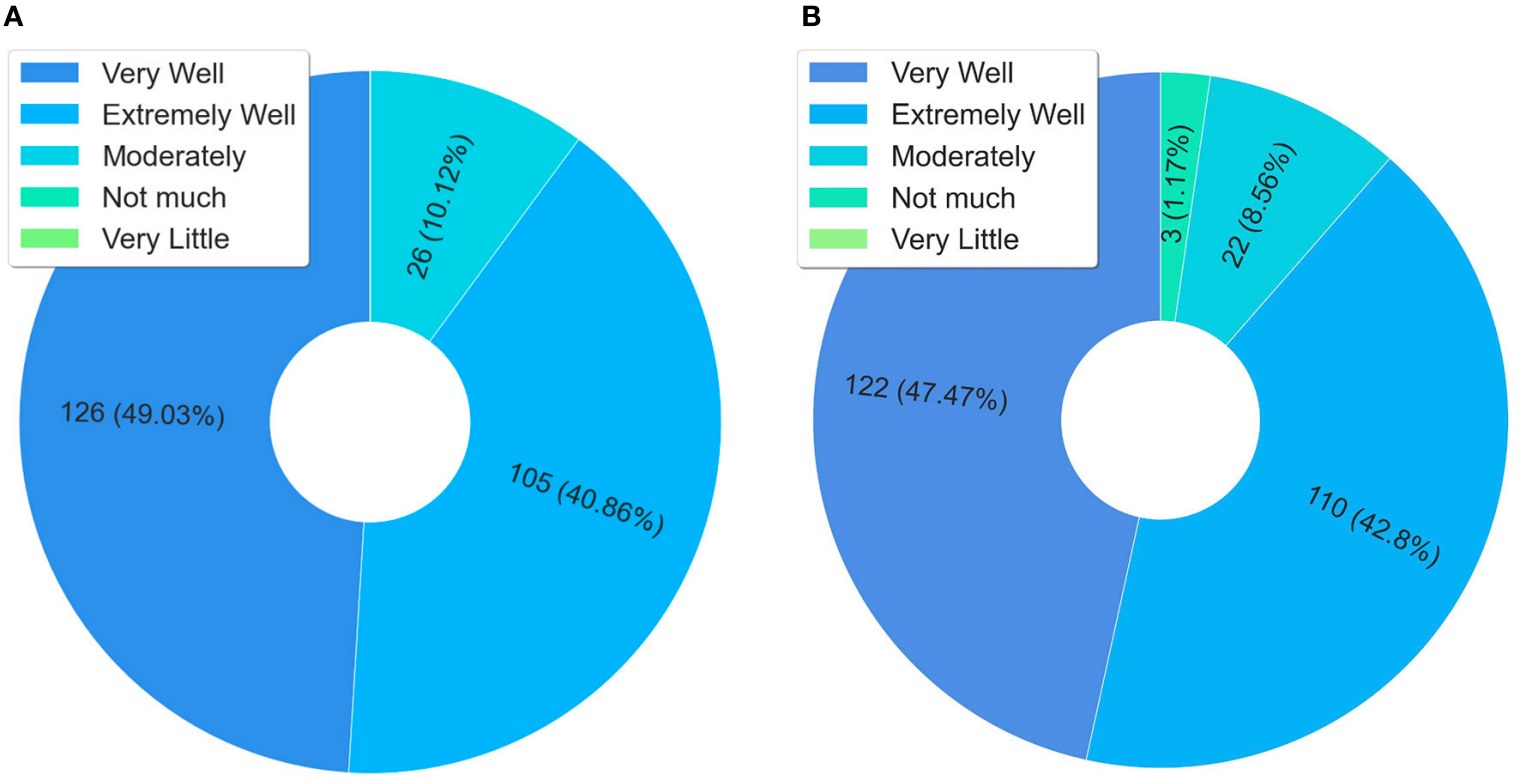
Analysis of content, epidemiological profile, and STIs. **(A)** Did the contents covered in the course discuss the epidemiological profile of the Brazilian prison system. **(B)** Did the course cover topics that help in the control of sexually transmitted infections in the prison system.

For ~90% of the questionnaire respondents, the course content addressed the epidemiological profile of the Brazilian prison system, according to the graph in Figure 5A. This same percentage was perceived in the graph of Figure 5B, when also, ~90% of these respondents, considered that the contents approach assistance in sexually transmitted infections control in the prison system.

**Figure 6:**
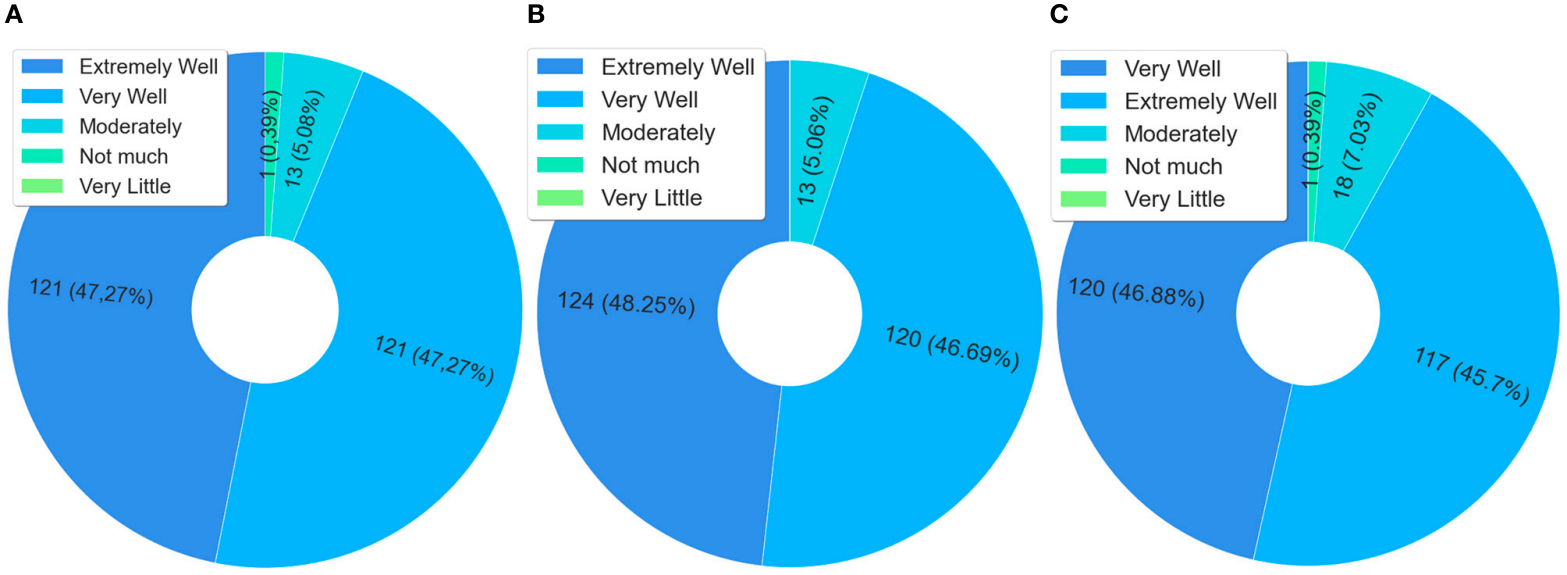
Analysis of comprehensive health care in the prison system. **(A)** Did the contents of the course cover effective strategies for comprehensive health care in the prison system? **(B)** Did the topics addressed in the course allow you to UNDERSTAND the aspects related to comprehensive care for “the health of men deprived of liberty”? **(C)** Did the topics addressed in the course allow you to UNDERSTAND the aspects related to comprehensive care for “the health of women deprived of liberty”?

The questions related to comprehensive health care in the prison system were grouped in Figure 6, so that it was possible to observe the students' perception with the health of men and women deprived of liberty. In the three scenarios of Figures 6A–C, more than 90% of the students who answered the questionnaire consider that the course contents address effective strategies for integral care. It is observed in the graph of Figures 6B, C that, according to these same students, these contents allowed an understanding of the aspects related to the integral care of men and women deprived of liberty.

**Figure 7:**
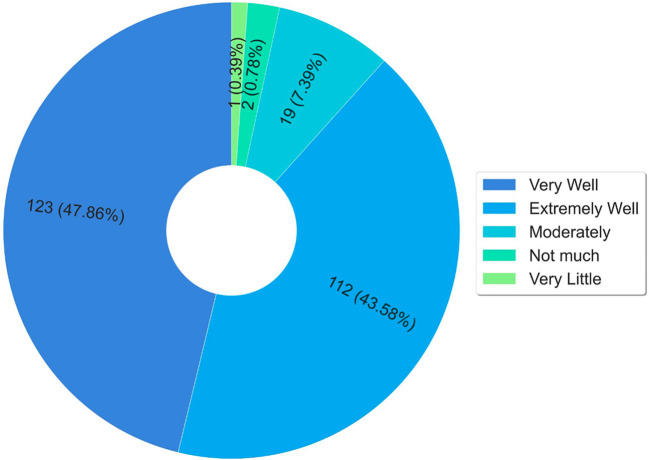
Mental health of the person deprived of liberty.

Figure 7 presents data related to the content and understanding of issues related to the mental health of detainees. In this case, when observing the responses of students who scored “extremely well” and “very well”, >91% considered that the course allowed them to understand the issues related to the mental health of detainees. It is also noteworthy that mental health in the prison system is also considered a public health problem, not only in Brazil but in the world (76). Therefore, this data helps to explain the meaning of the course content for the students, which also explains the issue of knowledge sharing.

**Figure 8:**
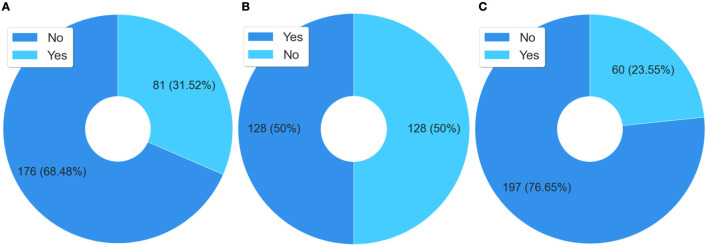
Analysis of students' previous knowledge. **(A)** Previous to the course, had you studied the content of the National Policy for Comprehensive Health Care for persons Deprived of Liberty in the Prison System (PNAISP)? **(B)** Previous to the course, had you studied the content of the National Policy for Integral Care of Women's Health (PNAISM)? **(C)** Previous to the course, had you studied the content of the National Policy for the Care of Women Deprived of Liberty and Released from the Prison System (PNAMPE)?

Figure 8 presents a summary of the results obtained about knowledge of National Policies. The data presented in the graph of Figure 8A show that 68.48% of the respondents did not know the national policy for comprehensive health care for people deprived of liberty before the course. The graph in Figure 8B highlights that 50% did not know about the national policy for women's health care before the course. The highest percentage of ignorance before the course was concerning the national policy for women deprived of liberty and egressed, which was 76.65%.

The findings presented in Figure 8 demonstrate the importance of this course for the training of professionals working in prison health in Brazil, since at least 50% of the respondents reported a lack of knowledge about the policies related to health care for people deprived of liberty.

In the last question of the dimension, students were invited to answer: “Which answer best fits your level of satisfaction with the course?”. The results obtained show that 99.22% are satisfied with the course, this is therefore another indicator of course quality.

The questionnaire respondents, when asked if, “Could improving health care in the prison system, observing the physical, psychological and social needs of people deprived of liberty, have a positive impact on society as a whole?” 99.21% totally agreed or agreed that yes. These data highlight the student's perception of the relevance of promoting integral care in prison health, and that this is related to society, i.e., they are inseparable in the respondents' perception - so it is possible to observe that in the respondents' general view, taking care of health in prisons is also taking care of society.

**Figure 9:**
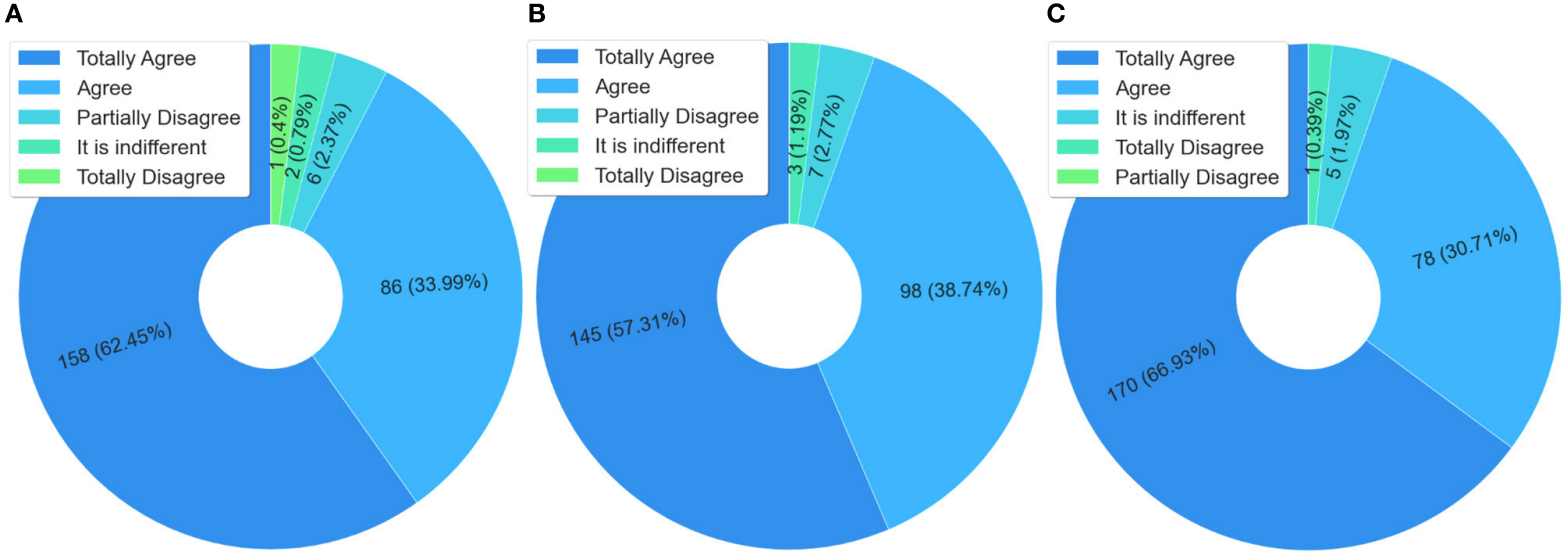
Analysis of prison health, diseases, community, and humanization. **(A)** Are deprivation of liberty and performance in the prison system vulnerability factor that intensify the appearance of various disease? **(B)** Is the restriction of comprehensive health care in the prison system a factor that intensifies the appearance of various diseases among people deprived of liberty and also in the community? **(C)** Does the improvement of health care in the prison system represent an advance toward a more adequate social and health system because it is more humanized?

Figure 9 presents the compilation of the answers to the questions that deal with deprivation of freedom and the association with diseases, in addition to presenting the perception of the respondents of the questionnaire regarding the improvement of health care with social and humanization issues. In this case, 96.44% of the respondents (Figure 9A) agree that the deprivation of freedom and the permanence in the prison system are vulnerability factors that favor getting sick. Figure 9B shows that 96.05% of the respondents also agree that the restriction to health in the prison system is another factor that favors the onset of diseases in the prison system. Regarding the improvement of prison health, 97.64% of respondents agree that this represents a social advance, because it means a more appropriate and humane treatment for detainees.

In the context of improving health in the prison system, 91% of the respondents totally agree or agree that it is possible to always achieve better levels of guaranteeing the right to health of the person deprived of liberty, according to the answers to the question “Is it possible to always achieve better levels of guarantee to the right to health of the person deprived of liberty?” This is positive because in their perception it is possible to develop a process of continuous improvement in prison health in Brazil.

**Figure 10:**
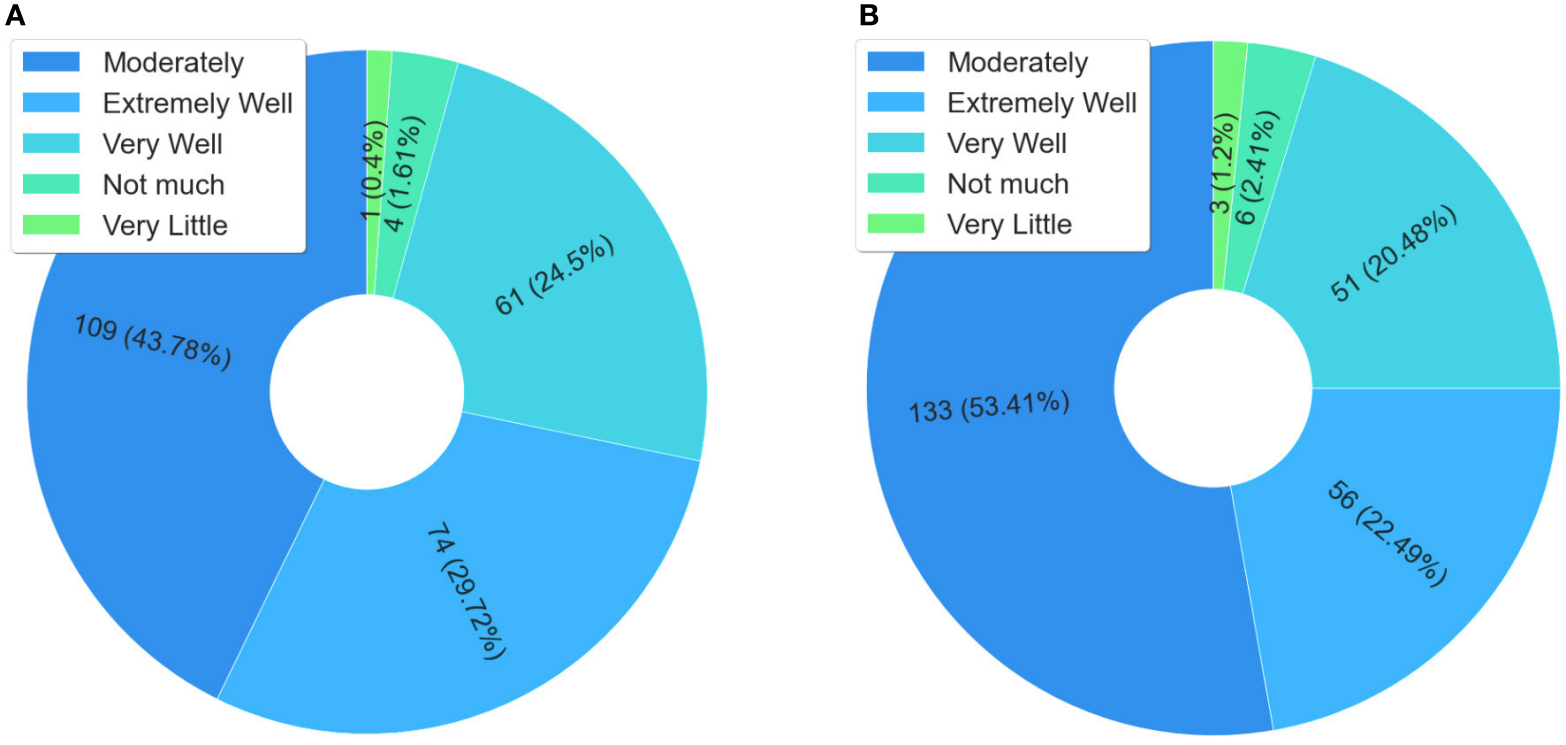
Analysis of improvements and behavioral changes in professional practice. **(A)** Did the course help to improve your professional practice? **(B)** Did the course provide change in behavior in your professional practice?

Regarding professional practice, it is possible to observe in Figure 10A, that 98% of those who answered the questionnaire considered that the course contributed to improving their professional practice. This is an important marker of quality, as it has a direct impact on the integral health care of people deprived of liberty, that is, on prison health.

Figure 10B shows the results from the following question, “Did the course help to improve your professional practice?”. For 96.38%, the course enabled a change in professional practice. This question is related to the previous one, and when observed together, can help explain changes in the behavior of professionals that can induce changes in the work processes, something desired when it is necessary to make interventions to improve health services—in this case through massive health education applied in the prison system.

**Figure 11:**
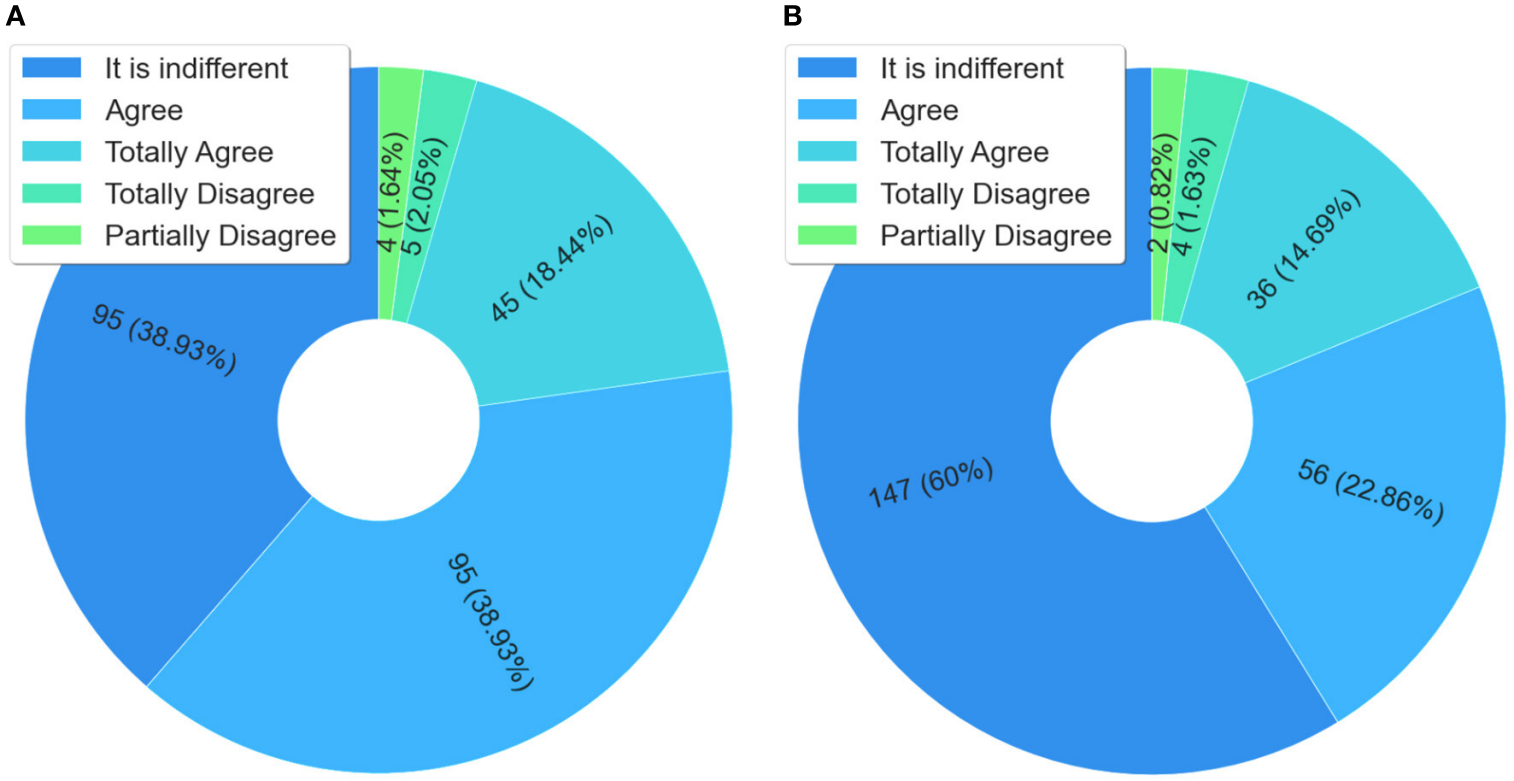
Analysis related to service improvement and work process changes. **(A)** Did the course improve an existing service? **(B)** Did the course provide change in the work process in the place where you work?

Regarding the work environment, Figure 11 shows that the course allowed enhancing an existing health service and whether it provided an opportunity for changes in the workplace. Note that for 57.37% of the respondents, Figure 11A, the course allowed the improvement of an existing health service, and for 37.55%, Figure 11B, the course induced changes in the work process at the place where the health professional (who was a student in the course) works. Thus, it presents the explicit evidence that demonstrates how massive health education, through technological mediation, is a viable tool for the intervention and induction of public policies in prison health.

The data from the answers to the question “Did the course provide changes in behavior in your professional practice?” indicate that, for more than 74% of the respondents, the course contributed to improving the welcoming and health care in the prison system in Brazil. It should be noted that improving the reception of prisoners means a change of behavior in the prison system, which also implies in the humanization of care in an environment where this aspect is often neglected. Therefore, this is a finding that helps to understand the impacts of health education in the prison system, because the humanized health care of detainees is a factor that indicates and improvement and enhancement of comprehensive health care in the prison system in Brazil.

## 4. Discussion

The results section presented several findings, essentially, from the perspective of students of the course “Health Care for People Deprived of Freedom”, who answered a questionnaire. It is noteworthy that the findings come from 246 (91.11%) of the 270 students who answered the questionnaire, showing that they work in some team of prison primary care. The data presented allowed answering the research question Q1: “How has massive education, mediated by technology, contributed to prison health in Brazil?”. Furthermore, relevant information was presented for the understanding of the respondents' universe, especially, when observing their area of training at the time they took the course. The data and information described in the results section allowed: a) to validate the application phase of the questionnaire as an evaluation tool for massive education. Through it, a positive spontaneous adhesion of students to a non-mandatory open course was verified, and, b) demonstrated that the search for the course is made by several areas of knowledge. In this case, there is a higher incidence of health professionals, but also of professionals in the humanities, who work directly with the guarantee of the rights of people deprived of liberty, for example, professionals in the areas of social worker, psychology, and education.

Before deepening the discussion it is important to highlight some characteristics of Brazil, once it is a country of continental dimensions with 26 federal units and the Federal District, more than 5,700 municipalities, and more than 3.5 million health professionals working in various areas (36). Still in this context, it is worth mentioning the social inequalities, besides having one of the largest prison populations in the world, which suffer from lack of adequate structure, overcrowding, and disease (53).

It is worth noting that students enrolled in the course “Health Care for People Deprived of Freedom” hailed from all regions of Brazil. Thus, 32.59% are from the Northeast Region, 32.88% from the Southeast, 18% from the South, 8.68% from the Midwest Region, 7.49% from the North, and 0.35% from other countries. The Southeast, Northeast, and South regions have the highest number of students and correspond to the regions with the highest prison population (1).

Another aspect observed was the level of education of the students enrolled. Despite being a free and open course, the target audience was primarily health professionals. This may help to explain the higher prevalence of such a student profile among enrollees. Therefore, this profile meets the necessary digital literacy requirements for AVASUS and has an education level that aligns with the course's purpose. This is mainly because such students have experience working in public health in Brazil. Hence, these constitute basic professional requirements.

Following this context, the development of a continuing education policy focused on prison health based only on the face-to-face model may be not only insufficient but also unfeasible from the perspective of cost-effectiveness that involves programs of this type in a country with the characteristics of Brazil. As a response to this challenge, the massive education of health professionals, through models whose educational architecture is directed by technological mediation, assumes a fundamental role. Therefore, according to the results presented, and considering that the course “Health Care for People Deprived of Freedom” on AVASUS has reached more than 11,500 enrollments throughout the country and that a significant number of these enrolled students have direct links with Primary Health Care in Prison, it is shown that this model was important to give capillarity and scalability to the course. This aspect favored the education of professionals working in prison health throughout Brazil - something that would be more difficult to achieve in the face-to-face model. However, if course enrollment were mandatory for all health professionals, particularly those working or wishing to work in Brazil's prison system, it would indeed have achieved a larger number of enrolled students.

It is worth noting that given the interfederative nature of SUS, mandatory enrollment in the course could entail a complex and very bureaucratic process, as it would require approval by a higher council of SUS. It is called the tripartite council, consisting of Brazil's Ministry of Health, the National Council of Health Secretaries (CONASS), and the National Council of Municipal Health Secretariats (CONASEMS). Notably, such a complexity becomes even more pronounced because prison health has been considered neglected, despite being something foreseen by law in Brazil. Unfortunately, this agenda has not received priority within SUS management levels. Thus, the strategy of adopting a technology-mediated model with spontaneous participation was the most viable, as in addition to accelerating the training process, it was not necessary to undergo the bureaucratic flow of the tripartite, which could take years to approve or never be on the agenda for a vote.

For a better understanding of the impacts of this course in the context of the performance and the work environment, it is also necessary to consider the PNAISP. This policy regulates the types of prison health teams and the professionals who make up these teams, which are the most diverse because the focus is the comprehensive care for people deprived of liberty. In line with the results presented, ~90% of the health professionals who took the course work or have worked in prison primary care. This data becomes even more significant because it directly impacts all the prison health teams, especially because these students said it is important to share the knowledge acquired with co-workers, in addition to recommending the course to them. Therefore, it is possible to infer that the course analyzed, in addition to contributing toward improving prison primary care teams in Brazil, acted as an inducer to strengthen the PNAISP.

Regarding the massive education in prison health as a tool to induce changes in the work process, it is noteworthy that more than 96% of respondents said that the course contributed to promoting changes in their professional practices. And that it was also important to provide improvements in existing health services, besides creating new services. This finding is important to explain the increase in syphilis testing and diagnosis in the prison system in Brazil between the years 2018 and 2019 (1). This phenomenon of increased notifications of syphilis cases in the Brazilian prison system in this period, at first could seem something negative, however, it was actually a change in the work process, because the prison primary care began to perform more tests, and consequently to notify more cases. It is noteworthy that until 2016 syphilis in Brazil was considered a neglected disease, so it was not on the country's public health agenda. In this context, the massive education for the prison system, in addition to promoting at scale the education of thousands of health professionals throughout Brazil, was also, inducing resilience in prison health. This is because a positive correlation was observed between the increase in enrollment in the course “Health care for the person deprived of freedom” and the increase in testing, as described in Valentim et al. (1), an aspect that corroborates the answers given by the respondents.

Against this background, the main contribution of the “Health Care for People Deprived of Liberty” course to prison health was the increase in STIs screening and care. This was found through the analysis of secondary data from the epidemiological bulletin issued by Brazil's National Prison Department (DEPEN) (1). Data and analysis were confirmed through the questionnaire, especially with the responses provided, as shown in Figure 10. In this instance, respondents stated that course content contributed to STI control in prison settings. Therefore, the rise in screening after course provision indicates changes in work processes. The latter aspect was emphasized in the questionnaire responses (see Figure 10), with respondents stating that the course contributed to enhancing an existing health service and work processes.

Thus, public policymakers need to reflect on the necessity for permanent investment in public policies for health care in the prison system. The change in work processes and professional practice that has repercussions in better assistance is indeed something significant and has appeared, for example, in the increase in the diagnosis of syphilis in the prison system. However, this does not depend, exclusively, on health professionals. Therefore, it is necessary to consider not only a greater investment in the prison system but also to enhance these investments, so that greater effectiveness can be achieved. According to the answers given to the questionnaire, almost 80% of the respondents affirmed that they work in the Prison Primary Care, but in the teams that dedicate the least weekly workload, only 6 hours a week. This is explained by the low remuneration of these professionals, who accumulate more than one work bond, and therefore cannot dedicate a greater workload to prison health. Therefore, in addition to the permanent and massive education, which has proven to be effective in prison health, the formulators of public policies should also observe the wage appreciation of health professionals working in the prison system, workloads appropriate to their activities and physical and structural improvements in workplaces, which enable the implementation of best practices of health care for deprived of freedom.

Still in this context, it is valid to reinforce that it was in 2018 that the course “Health Care for People Deprived of Liberty” was made available in the Virtual Learning Environment of the Brazilian Health System, that is, one year after the beginning of the course, 2019, Brazil recorded the highest peak of syphilis testing in the prison system (1, 2, 51). Although the data do not demonstrate causality, they reinforce that continuing education can positively impact the health care and assistance services provided in the prison system (1). It is noteworthy that testing and diagnosis are premises for the treatment and cure of syphilis, and also to reduce the transmission curve of this infection in the prison system, objectives observed after the massive education process with technological mediation promoted by the course studied.

These aspects are shreds of evidence that help to demonstrate that massive health education with technological mediation is a relevant tool to be used to drive public health policies, specifically in neglected fields such as prison health (77). This was even more apparent when we considered that the interest in the course came about spontaneously. In this respect, it is worth noting that massive health education becomes even more effective, especially in times of health crises, as was recently seen with the COVID-19 pandemic (41, 78). This is because it is necessary to upskill the workforce and health services to respond more quickly to public health emergencies, aiming to promote and instill resilience in the health system. In this regard, Henriques et al. (79) underscore the need to revisit the debates on the interactions between technology and education and the added value of digital resources to enhance educational processes.

For that reason, the biggest challenge in implementing this massive education program–mainly as it is situated in the context of prison health–is the lack of prioritization. Unfortunately, prison health continues to be overlooked in Brazil. This is an important constraint to improving health in the prison system. Therefore, it is necessary to encourage the development of public policies in the context of prison health, in addition to sustaining and fostering continuing education programs that can work toward improving the four pillars of the prison system: health professionals, people deprived of liberty, prison officers, and managers. A shortcoming of the massive education process is the need for investment in adequate infrastructure, as it is a technology-mediated process. Nonetheless, in countries with Brazil's dimensions and a very high prison population, such a model can be viable due to its cost-effectiveness and the need for a more timely response.

## 5. Conclusions

Evaluating massive Education in Prison Health is a strategic activity to think about the contributions and impacts of this training on the National Policy of Continuing Health Education (80, 81) and the strengthening of technology-mediated education. In the specific case, this research observed and analyzed the contributions in the context of health in the prison system in Brazil, especially, the positive impacts on the improvement of the health workforce, health services, and epidemiological indicators of prison health. However, beyond these contributions, the massive education in prison health, also contributed transversally to the social agenda of the United Nations Educational, Scientific and Cultural Organization (UNESCO). This agenda sets a new social contract for global education, a fundamental aspect, because it is a repairing agent that acts to reduce injustices and iniquities, therefore transforming the future (82).

From this perspective, the relevance of massive education for prison health crosses the improvement of the supply of health services in the prison environment and reaches the Sustainable Development Goals (SDGs) of UN's 2030 Agenda (26, 83). It is noteworthy that the main motto of this agenda is “Leave no one behind (LNOB)”, based on five fundamental principles: people, planet, prosperity, peace, and partnerships, which form the five pillars of the SDGs (83–85). When analyzing the contributions, impacts and nature of the course “Health Care for People Deprived of Liberty” and based on the answers obtained through the questionnaire applied, it is possible to identify a greater emphasis on the following SDGs: 3, 4, 10, 16 and 17.

The cited goals showed that massive Education in Prison Health can be situated in an Educational Pentagon that addresses the dimensions: health, education, combating inequalities, peace and justice in institutions and partnerships toward the goals and sustainable development for all people. It is noteworthy that SDG 3 is concerned with ensuring a healthy life and promoting well-being for all, this aspect was observed in the analysis of the answers given by health professionals working in the Brazilian prison system. SDG 4, deals with inclusive, equitable, and quality education, promoting lifelong learning opportunities for all. Therefore, by using technological mediation to ensure the greatest possible access to knowledge (scalability), the course studied was able to reach all levels of health care in the Brazilian prison system. Moreover, the evaluations highlighted by the students who answered the questionnaire demonstrated the quality of the course.

Regarding SDG 10, in favor of reducing inequality within and between countries, it is observable from the analysis developed, that the promotion of prison health significantly reduces inequalities, with greater repercussion in the prison system, by ensuring both the right to health and allow continuing health education through technological mediation. For SDG 16, whose goal is to promote peaceful and inclusive societies for sustainable development, provide fair access to rights for all people. It is noticed that the massive education in prison health contributes to the improvement of a more humanized health care in the prison system, an aspect that reverberates to a more peaceful society and collaborates with the effectiveness of the institutions, in guaranteeing rights through education and health - taking care of prison health is taking care of society. Based on SDG 17, the course, “Health Care for People Deprived of Liberty”, acts in the implementation and revitalization of global partnerships for sustainable development. Therefore, it is observed the contributions of massive education in prison health as an articulator of public health policies, permanent education policies, and the law of penal execution. A network of institutional cooperation, involving the Brazilian Ministry of Health, the Brazilian Ministry of Education and the Brazilian Ministry of Justice, in a pact for those deprived of freedom and with the enhanced support of professionals in the prison system.

The best expression for the analysis of massive education in prison health is the effectiveness of its open educational resources (OER), which impact on the improvement, not only of people in the context of deprivation of liberty, but also in building a society and a planet more just, peaceful and prosperous. Therefore, it is necessary to think that this Educational Pentagon can be a resilience-inducing tool, capable of reaching all of society, so that it can act in a more improved manner in the development as freedom, capable of mitigating the effects of injustices that may affect the global future (86).

## 6. Limitations

This research has drawn on the analysis of a questionnaire applied to participants of the course “Health Care for People Deprived of Freedom,” offered through the Virtual Learning Environment of the Brazilian Health System (AVASUS). Questionnaire respondents were primarily practicing in healthcare and primary care in prisons. That represents the main limitation of our study, as it has not been possible to verify the impacts of the health education process from the perspective of other actors included in the prison system, such as prison officers, people deprived of their liberty, and managers. Since this was not the aim of this study, future research may address this issue.

## Data availability statement

The datasets presented in this study can be found in online repositories. The names of the repository/repositories and accession number(s) can be found below: https://doi.org/10.5281/zenodo.8034239.

## Author contributions

JV, SD-T, MRo, EO, RM, and RV contributed to conception and design of the study. FF, PM, and KM organized the database and repository. JV, MRo, FF, AC, GF, and RV performed the analysis. JV, MRo, AC, MRo, CG, and RV wrote the first draft of the manuscript. JV, MRo, AC, and RV wrote sections of the manuscript. All authors contributed to manuscript revision, read, and approved the submitted version.
